# Supplementary material for: The High Capacity of Brazilian Aedes aegypti Populations to Transmit a Locally Circulating Lineage of Chikungunya Virus
Source: Viruses. 2024 Apr 9;16(4):575. doi: 10.3390/v16040575 (PMC11053879; doi:10.3390/v16040575)
Supplement: Supplementary file 1 [file viruses-16-00575-s001.zip › Table S1.pdf]

Reserved space. Do not place any text in this section. Include the mandatory author checklist or your manuscript will be returned.

Table S1. Summary of Logistic Regression Model for CHIKV Infection Rates.

4 days post feeding

**Predictors**	**Category**	**Reference**	***Odds Ratios***	***CI (95%)***	***P-value (Wald´s test)***	***Overall P-value (LR-test)***
	(Intercept)		0.16	0.04 – 0.46	0.003	
Population	JAB	ARA	0.00	NA	0.994	0.2093
	PET	ARA	0.63	0.08 – 4.23	0.637	
	POA	ARA	0.63	0.08 – 4.23	0.637	

Observations 88
R^2 Tjur 0.034

8 days post feeding

**Predictors**	**Category**	**Reference**	***Odds Ratios***	***CI (95%)***	***P-value (Wald´s test)***	***Overall P-value (LR-test)***
	(Intercept)		0.05	0.00 – 0.23	0.003	
Population	JAB	ARA	3.32	0.39 – 70.24	0.317	0.5197
	PET	ARA	3.32	0.39 – 70.24	0.317	
	POA	ARA	4.67	0.62 – 95.79	0.185	

Observations 88
R^2 Tjur 0.022

4 and 8 days post feeding

**Predictors**	**Category**	**Reference**	***Odds Ratios***	***CI (95%)***	***P-value (Wald´s test)***	***Overall P-value (LR-test)***
	(Intercept)		0.10	0.03 – 0.25	<0.001	
Population	JAB	ARA	0.73	0.14 – 3.52	0.695	0.7393
	PET	ARA	1.28	0.32 – 5.51	0.725	
	POA	ARA	1.58	0.42 – 6.59	0.504	

Observations 176
R^2 Tjur 0.007
