# Supplementary material for: The High Capacity of Brazilian Aedes aegypti Populations to Transmit a Locally Circulating Lineage of Chikungunya Virus
Source: Viruses. 2024 Apr 9;16(4):575. doi: 10.3390/v16040575 (PMC11053879; doi:10.3390/v16040575)
Supplement: Supplementary file 1 [file viruses-16-00575-s001.zip › Table S2.pdf]

Reserved space. Do not place any text in this section. Include the mandatory author checklist or your manuscript will be returned.

Table S2. Summary of Logistic Regression Model for CHIKV Dissemination Efficiency.

4 days post feeding

**Predictors**	**Category**	**Reference**	***Odds Ratios***	***CI (95%)***	***P-value (Wald´s test)***	***Overall P-value (LR-test)***
	(Intercept)		0.05	0.040 – 0.24	0.003	
Population	JAB	ARA	2.00	0.18 – 45.09	0.584	0.012
	PET	ARA	2.22	0.20 – 50.23	0.529	
	POA	ARA	3.16	0.37 – 66.97	0.337	

Observations 85
R^2 Tjur 0.012

8 days post feeding

**Predictors**	**Category**	**Reference**	***Odds Ratios***	***CI (95%)***	***P-value (Wald´s test)***	***Overall P-value (LR-test)***
	(Intercept)		0.10	0.02 – 0.34	0.002	
Population	JAB	ARA	1.00	0.11 – 9.01	1.000	0.6602
	PET	ARA	1.00	0.11 – 9.01	1.000	
	POA	ARA	2.50	0.43 – 19.74	0.324	

Observations 86
R^2 Tjur 0.021

4 and 8 days post feeding

**Predictors**	**Category**	**Reference**	***Odds Ratios***	***CI (95%)***	***P-value (Wald´s test)***	***Overall P-value (LR-test)***
	(Intercept)		0.08	0.02 – 0.21	<0.001	
Population	JAB	ARA	1.33	0.28 – 7.13	0.718	0.5226
	PET	ARA	1.40	0.29 – 7.52	0.670	
	POA	ARA	2.67	0.68 – 10.10	0.178	

Observations 171
R^2 Tjur 0.014
