# Supplementary material for: The High Capacity of Brazilian Aedes aegypti Populations to Transmit a Locally Circulating Lineage of Chikungunya Virus
Source: Viruses. 2024 Apr 9;16(4):575. doi: 10.3390/v16040575 (PMC11053879; doi:10.3390/v16040575)
Supplement: Supplementary file 1 [file viruses-16-00575-s001.zip › Table S3.pdf]

Reserved space. Do not place any text in this section. Include the mandatory author checklist or your manuscript will be returned.

Table S3. Summary of Logistic Regression Model for CHIKV Transmission Efficiency.

8 days post feeding

**Predictors**	**Category**	**Reference**	***Odds Ratios***	***CI (95%)***	***P-value (Wald´s test)***	***Overall P-value (LR-test)***
	(Intercept)		0.25	0.040 – 1.00	0.080	
Population	JAB	ARA	1.33	0.13 – 13.97	0.800	0.9302
	PET	ARA	2.00	0.25 – 19.18	0.513	
	POA	ARA	1.50	0.20 – 13.86	0.697	

Observations 38
R^2 Tjur 0.012
